# A green strategy to produce potential substitute resource for bear bile using engineered *Saccharomyces cerevisiae*

**DOI:** 10.1186/s40643-022-00517-3

**Published:** 2022-03-27

**Authors:** Lina Jin, Li Yang, Shujuan Zhao, Zhengtao Wang

**Affiliations:** The SATCM Key Laboratory for New Resources & Quality Evaluation of Chinese Medicine, The MOE Key Laboratory for Standardization of Chinese Medicines and Shanghai Key Laboratory of Compound Chinese Medicines, Institute of Chinese Materia Medica, Shanghai University of Traditional Chinese Medicine, Shanghai, 201203 People’s Republic of China

**Keywords:** Tauroursodeoxycholic acid, Hydroxysteroid dehydrogenase, Biotransformation, *Saccharomyces cerevisiae*

## Abstract

**Background:**

Bear bile powder is a precious natural material characterized by high content of tauroursodeoxycholic acid (TUDCA) at a ratio of 1.00–1.50 to taurochenodeoxycholic acid (TCDCA).

**Results:**

In this study, we use the crude enzymes from engineered *Saccharomyces cerevisiae* to directionally convert TCDCA from chicken bile powder to TUDCA at the committed ratio in vitro. This *S. cerevisiae* strain was modified with heterologous 7α-hydroxysteroid dehydrogenase (7α-HSDH) and 7β-hydroxysteroid dehydrogenase (7β-HSDH) genes. *S. cerevisiae* host and HSDH gene combinatorial optimization and response surface methodology was applied to get the best engineered strain and the optimal biotransformation condition, respectively, under which 10.99 ± 0.16 g/L of powder products containing 36.73 ± 6.68% of TUDCA and 28.22 ± 6.05% of TCDCA were obtained using 12.00 g/L of chicken bile powder as substrate.

**Conclusion:**

This study provides a healthy and environmentally friendly way to produce potential alternative resource for bear bile powder from cheap and readily available chicken bile powder, and also gives a reference for the green manufacturing of other rare and endangered animal-derived valuable resource.

## Introduction

Bear bile powder is a kind of precious material that has been used as medicine and healthcare supplementary thousands of years ago in Asia area (Yang et al. 2008; Feng et al. 2009). It has multiple pharmacological activities and can be used to treat gallstones, cholecystitis, fatty liver and other hepatobiliary diseases (Feng et al. 2009). At present, bear bile powder is mainly from the drainage bear bile extracted from the farmed living bears including *Selenarctos thibetanus* (Asiatic black bear) or *Ursus arctos* (brown bear) with “Free-dripping Fistula Technique” by implanting a duct or making an artificial fistula in the liver of the bears (Feng et al. 2009). These methods will seriously endanger the health of bears, thus exploring substitute resources is of great significance to meet the demands of bear bile powder as medicinal and healthcare material.

Bile acids (BAs), a group of steroids with C-17 side chains, are the principal bioactive ingredients of bear bile powder, among which ursodesoxycholic acid (UDCA) or its physiologically active form tauroursodeoxycholic acid (TUDCA) takes a high proportion and is considered as distinct and character of bear bile from other animal bile (Ferrandi et al. 2012; Eggert et al. 2014; Zheng et al. 2017). The authentication and standard quality work from 20 batches of drainage bear bile powder samples in our lab revealed that the average content of TUDCA was 26.50%, and the ratio of TUDCA to TCDCA (TUDCA/TCDCA) was from 1.00: 1.00 to 1.50: 1.00 (Wang et al. 2018), which could be regarded as the characteristics of bear bile powder.

Till now, the synthesized TUDCA and UDCA are the only acceptable substitutes for bear bile because it has similar bioactivities as bear bile, including neuroprotective action, promoting pancreatic survival and function, and reducing gallstone formation (Lu et al. 2021; Rosa et al. 2021; Zangerolamo et al. 2021). There are several methods to produce UDCA or TUDCA such as chemical synthesis, whole-cell biocatalysts, and the chemo-enzymatic method (Momose et al. 1997; Liu et al. 2013; Zheng et al. 2015). All these methods required pure and rare compounds as precursors or substrates, which increased the production costs and limited the industrial application. In addition, bile acids other than TUDCA or UDCA also have biological activities. For example, TCDCA had anti-inflammatory effect, could stimulate intestinal cell proliferation, protect against apoptotic cell death, and alleviate pulmonary fibrosis (Toledo et al. 2004; Zhou et al. 2013; Li et al. 2019). Therefore, it would be more feasible to explore alternatives based on both the therapeutic effects and the specific chemical properties of natural bear bile powder.

**Fig.1:**
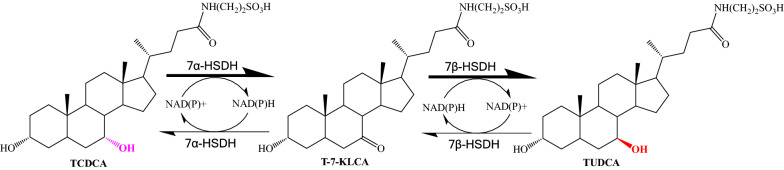
Reaction catalyzed by 7α-HSDH and 7β-HSDH

Different from bear bile, poultry bile such as chicken bile is a cheap and easily available resource. It contains high amount of TCDCA but no TUDCA. Chemically, TUDCA is the epimer of TCDCA at C-7 hydroxyl. Bacterial 7α-hydroxysteroid dehydrogenase (7α-HSDH) and 7β-hydroxysteroid dehydrogenase (7β-HSDH) have both oxidative and reductive activities and can interconvert TCDCA and TUDCA coupling with NAD^+^ or NADP^+^ as co-factor (Yoshimoto et al. 1991; Ferrandi et al. 2012; Lee et al. 2013; Song et al. 2017) (Fig. 1). We had constructed an engineered *Escherichia coli* with 7α-HSDH and 7β-HSDH genes which could convert a certain proportion of TCDCA to TUDCA using chicken bile powder as substrates.

As a safe and health microbial organism with clear genetic background, *Saccharomyces cerevisiae* has been widely used in food and pharmaceutical industry (Lian et al. 2018). Here, taking the advantage of *S. cerevisiae* and the bi-directional catalytic properties of HSDH, we created an efficient way to produce the potential substitute for bear bile powder using chicken bile powder as raw material through the engineered *S. cerevisiae*. To our knowledge, this is the first report of applying engineered *S. cerevisiae* to make products having certain amount of TUDCA and TCDCA equivalent to that in bear bile powder.

## Materials and methods

### Chemicals and reagents

Chicken bile powder (containing 64.78 ± 0.30% of TCDCA) were kindly provided by Shanghai Kaibao Pharmaceutica Co. Ltd., China. Resin D101 was purchased from Wuhan Weiqiboxin Biotechnology Co. Ltd., China. β-NAD^+^ and NADP Na_2_ was purchased from Coolaber Science & Technology (Beijing, China). All the other chemicals in this study were from Sinopharm Chemical Reagent Co. Ltd., China.

### Gene selection, codon optimization and synthesis

Two 7α-HSDH and two 7β-HSDH genes originating from *Clostridium sardiniense*, *E. coli*, and *Ruminococcus gnavus* (GenBank accession numbers JN191345, D10497, and KF052988) were selected to construct four combinations as our previous work (Shi et al. 2017). Briefly, the coding region of these genes was codon optimized and synthesized by Life Technologies (Shanghai, China), named as Ca7α-HSDH^syn^, Ec7α-HSDH^syn^, Ca7β-HSDH^syn^, and Rg7β-HSDH^syn^ (GenBank accession numbers KY178305-KY178308) referred to as α1, α2, β1, and β2, respectively.

### Microbial strains, plasmids, and expression vector construction

Two *S. cerevisiae* strains W303-1a (MATa; ade2-1; ura3-1; his3-11; trp1-1; leu2-3; leu2-112; can1-100) and CEN.PK2-1C (MATa; his3D1; leu2-3_112; ura3-52; trp1-289; MAL2-8c; SUC2) were used as the hosts to expression the 7α-HSDH^syn^ and 7β-HSDH genes. *E. coli* DH5α was used as hosts for middle expression vectors. Yeast shuttle plasmids pRS424 and pRS426 were separately used to carry 7α-HSDH^syn^ and 7β-HSDH^syn^ expression cassettes assembled from P_PGK1_, 7α-HSDH^syn^ and T_PGI_, or P_TDH3_, 7β-HSDH^syn^ and T_PDC1_, respectively. A total of four yeast expression vectors were generated, namely pRS424-P_PGK1_-α1-T_PGI_, pRS424-P_PGK1_-α2-T_PGI_, pRS425-P_TDH3_-β1-T_PDC1_, and pRS425-P_TDH3_-β2-T_PDC1_. One 7α-HSDH^syn^ combined with one 7β-HSDH^syn^ expression vectors were co-introduced into *S. cerevisiae* strains W303-1a and CEN.PK2-1C, yielding eight engineered yeast strains, named W303a-α1β1, W303a-α1β2, W303a-α2β1, W303a-α2β2, CEN-α1β1, CEN-α1β2, CEN-α2β1, and CEN-α2β2.

### Cultivation of the engineered *S. cerevisiae* strains

The engineered yeast cells were cultured in flasks containing SD-Trp^−^-Ura^−^ liquid media at 30^◦^C, 220 rpm on a horizontal shaker. For engineered strain screening, a single colony of each strain was inoculated in 25 mL of liquid media and cultured for 24 h. Then the yeast cell cultures were sub-cultured in 400 mL of liquid media at a proportion of 1:20 and grew for another 24 h. For biotransformation condition optimization, another scale-up sub-culture process was carried out in 8 L of liquid media using the first sub-cultures as seed cells at the same ratio for the third 24 h. The yeast cell cultures were collected to get the cells for crude enzyme preparation.

### Crude enzyme preparation

The engineered *S. cerevisiae* cells were collected by centrifugation. The cells were washed with sterile water and phosphate buffer saline (PBS, 100 mM) twice, and resuspended in appropriate volume of PBS. Cells were lysed either by 10 cycles of vortex-ice bath (vortex 30 s, ice bath keeping for 30 s) after adding certain amount glass beads (diameter: 424–600 μm), or by homogenization under 600–1000 bar. The lysed cell mixture was centrifuged at 4 °C, 12,000 rpm for 5–10 min, and the supernatant that was the crude enzyme solution was transferred into new tubes. The crude enzyme powder was obtained by drying in a freeze dryer. For engineered yeast strain screening, 10 mL of cell cultures were finally resuspended in 150 μL of PBS. For biotransformation condition optimization, 8 L of cell cultures were finally resuspended in 200 mL of PBS, and the concentration of total proteins was measured with Braford method and calculated as 1.08 ± 0.02 mg/mL.

### In vitro biotransformation of TUDCA

The biotransformation of TUDCA was conducted in a reaction mixture comprising 100 mM of PBS, a certain amount of crude enzyme solution and chicken bile powder. For strain screening and biotransformation condition optimization, the reaction was carried out in 1 mL reaction mixture (pH 6.5) with 0.10 g/L of NADP Na_2_, 150 µL of crude enzyme solution, and 4.80 g/L of chicken bile powder at 30 °C for 6 h, refer to our previous work (Shi et al. 2017; Xu et al. 2019). For product preparation, the reaction mixture (pH 7.0) was 1 L containing 170 mL of crude enzyme solution and 12.00 g/L of chicken bile powder and biotransformation condition was 25 °C for 5.23 h. After incubation, the reaction was stopped by keeping in boiling water for 5 min, followed by centrifugation. The supernatant was either analyzed by HPLC or made to powder product. Concentration of TUDCA and the conversion efficiency was used to evaluate the production capacity of engineered *S. cerevisiae* strains. In this study, the conversion efficiency was indicated as the TUDCA yield calculating according to Eq. (1) as followed:

1 $${\text{Conversion efficiency}} = \frac{{{\text{Total amount of TUDCA in products }}\left( {\text{g}} \right)}}{{{\text{Total amount of TCDCA in substrates }}\left( {\text{g}} \right)}} \times 100\% .$$

### In vitro biotransformation condition optimization

Based on the catalytic properties of the original 7α-HSDH and 7β-HSDH, and our findings on *E. coli* (Shi et al. 2017; Xu et al. 2019), substrate concentration, pH value, temperature, and the fermentation time also have influence on the substrate conversion efficiency. Meanwhile, 7α-HSDH and 7β-HSDH are NAD(P)^+^-dependent enzymes. The supply of NADP Na_2_ in the reaction mixture would also have effect on the enzyme catalytic activity thereby affecting the conversion efficiency. Taken all these factors into account, in vitro biotransformation conditions were optimized through employing single factor exploration and response surface methodology (RSM) by setting a certain range of each factor.

The effects of substrate concentration, ranging from 2.40 g/L to 40.00 g/L, and pH value, covering 6.0 to 7.5, on the conversion were separately investigated. The influence of temperature, incubation time, and the concentration of NADP Na_2_ was evaluated using Behnken Design (BBD) of RSM. These three independent factors, respectively, designed as factor A, B, C were investigated at three different levels. Factor A was divided into 20 °C, 30 °C, and 40 °C, factor B was divided into 1 h, 5 h, and 9 h, and factor C was divided into 0.05 g/L, 0.10 g/L, and 0.15 g/L with five repetitions at the central point using the ratio of TUDCA/TCDCA as response. The data are analyzed by using Design-Expert.V8.0.6.1 software. For RSM experiment, 12.00 g/L of substrates and pH 7.0 was used according to results of single factor exploration.

### Preparation of powder products

Isometric resin D101 was used to purify the conversion products. To do that, the supernatant of the reaction mixture was passed through pre-treated isometric resin D101. The impurities on D101 resin were firstly removed by deionized water until the eluent was colorless. Then the resin was washed with 95% ethanol to get the eluent until colorless. The eluent was mixed and concentrated through rotary evaporation at 50 °C and then filtered through 0.45 μm organic filters. The filtrate was evaporated by keeping in water bath at 50 °C to form extractum and then dried to constant weight in vacuum drying oven at 50 °C. After that, the dried solid was taken out and crushed into powder that is the products.

### Bile acid analysis

For conversion efficiency assay, the supernatant was directly filtered through a 0.22 μm filter and supplied to the ThermoUltiMate3000 HPLC machine. For prepared product assay, methanol was added to resolve the dried bile acids and filtered through a 0.22 μm filter before supplied to HPLC machine. The concentration of individual bile acid was determined according to the curve of each authentic standard.

### HPLC condition

HPLC was performed according to our previous work (Xu et al. 2019). Briefly, a ThermoUltiMate3000 HPLC machine equipped with Agilent Poroshell 120 EC-C18 column (2.7 μm,4.6 mm × 150 mm) and Corona Ultra Detector (CAD) was employed using acetonitrile and water (containing 0.3% formic acid and 5 mM ammonium acetate) as the mobile phase at a flow rate of 0.6 mL/min. HPLC gradient elution program was total 45 min with acetonitrile proportion from 20 to 90%.

## Results

### High productive engineered *S. cerevisiae* strain screening

**Fig. 2:**
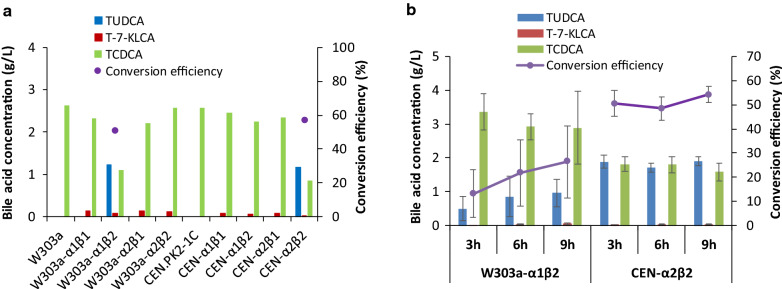
Results of high productive engineered *Saccharomyces* *cerevisiae* screening. **a** Results of eight engineered yeast strains and two control yeast strains under 30 °C, pH 6.5 for 6 h. **b** Results of W303a-α1β2 and CEN-α2β2 under 25 °C, pH 6.5

To screen a high productive engineered yeast strain, crude enzymes from the eight engineered *S. cerevisiae* strains were incubated with 4.80 g/L of chicken bile powder in PBS at 30 °C. After incubation for 6 h, only two strains, W303a-α1β2 and CEN-α2β2, produced TUDCA (Fig. 2a). The conversion efficiency of the two strains was 51.0% and 56.9% (Fig. 2a) and the ratio of TUDCA to TCDCA was 1.37 and 1.12, respectively. Meanwhile, there was a little of tauro-7-ketone lithocholic acid (T-7 K-LCA) intermediate built up in all those constructs. The conversion efficiency of crude enzymes from these two strains was further analyzed to select a more practicable one by swapping the biotransformation condition to 25 °C for 3 h, 6 h and 9 h. As shown in Fig. 2b, the conversion efficiency of CEN-α2β2 was 50.62 ± 5.45%, 48.44 ± 4.79%, and 54.18 ± 3.44% and the ratio of TUDCA to TCDCA was 1.05 ± 0.23, 0.97 ± 0.18, and 1.22 ± 0.17, respectively; whereas that of W303a-α1β2 was below 40% and 0.2, respectively. These results indicated that α2β2 has higher enzymatic catalytic capability than α1β2, possible due to the enzymatic activity of α2 was more flexible than that of α1 under the given conditions. It also indicated that incubation for 9 h was better. Therefore, CEN-α2β2 was selected in next experiments.

### Effect of substrate concentration on the biotransformation of TUDCA

**Fig. 3:**
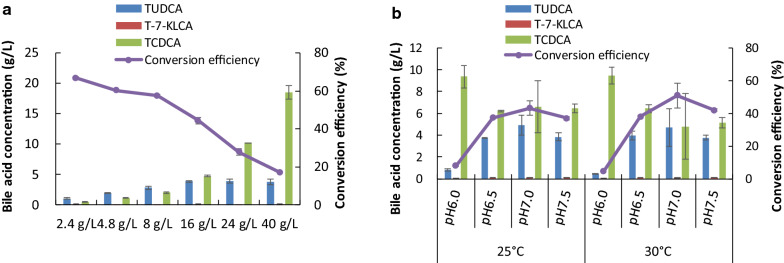
Results of substrate concentration and pH value on the biotransformation of TUDCA. **a** Results of substrate concentration when incubation at 30 °CC, pH6.5 for 9 h. **b** Results of pH value when incubation for 9 h

To screen an optimal substrate concentration, the reaction mixture was incubated at 30 °C, pH 6.5 for 9 h. As shown in Fig. 3a, the conversion efficiency ranged from 66.59 ± 0.93%, 60.22 ± 0.18%, 57.31 ± 0.71%, 44.32 ± 1.63%, 27.76 ± 1.64% to 17.01 ± 0.71% and the ratio of TUDCA/TCDCA was 2.31 ± 0.03, 1.63 ± 0.01, 1.39 ± 0.04, 0.81 ± 0.06, 0.39 ± 0.03, and 0.21 ± 0.01, when feeding with 2.40 g/L, 4.80 g/L, 8.00 g/L, 16.00 g/L, 24.00 g/L, and 40.00 g/L of substrate, respectively. This result suggested that the conversion efficiency decreased with the increase of substrate concentration under the given conditions, and 8.00–16.00 g/L of chicken bile powder was good for in vitro biotransformation.

### Effect of pH value on the biotransformation of TUDCA

The effect of pH value on TUDCA formation was assessed by setting the incubation at 25 °C or 30 °C for 9 h and feeding 16.00 g/L of chicken bile powder. As shown in Fig. 3b, with the increase of pH value, the yield of TUDCA increased at first and then decreased under both temperatures. When pH value was 7.0, the conversion efficiency was the highest, 43.05 ± 4.40% and 50.77 ± 7.34%, and the ratio of TUDCA to TCDCA was 0.77 ± 0.14 and 1.08 ± 0.32 at 25 °C and 30 °C, respectively. These results indicated that 30 °C combined with pH 7.0 was the best condition for the biotransformation, followed by 30 °C with pH 7.5, and 25 °C with pH 7.0, when incubation for 9 h.

### Evaluation of experimental design of Box–Behnken design of RSM

The results of RSM of 17 runs from Box–Behnken design (BBD) experiments to study the effects of the three independent factors, temperature (factor A), incubation time (factor B), and the concentration of NADP Na_2_ (factor C) on the ratio of TUDCA/TCDCA was loaded into Design-Expert.V8.0.6.1 software for regression analysis (Table 1). The quadratic polynomial regression equation describing the response value and independent variables is obtained as below:

2 $${\text{Y }}\left( {\frac{{{\text{TUDCA}}}}{{{\text{TCDCA}}}}} \right) = + { }1.01 - 0.21{\text{*A}} + 0.28{\text{*B}} + 0.17{\text{*C}} - 0.20{\text{*A*B}} - 7.500{\text{E}} - 003{\text{*A*C}} + 0.068{\text{*B*C}} - 0.22{\text{*A*A}} - 0.22{\text{*B*B}} - 0.055{\text{*C*C }}{.}$$

**Table 1:** Experimental designs and the results of the Box–Behnken design

No.	A-Temperature (°C)	B-Incubation time (h)	C-NADP Na_2_ (mg)	TUDCA/TCDCA
1	30.00	9.00	0.15	1.28
2	20.00	1.00	0.10	0.36
3	30.00	5.00	0.10	0.94
4	30.00	5.00	0.10	1.09
5	30.00	1.00	0.05	0.33
6	30.00	5.00	0.10	0.88
7	40.00	1.00	0.10	0.34
8	40.00	5.00	0.15	0.72
9	30.00	5.00	0.10	1.02
10	30.00	1.00	0.15	0.48
11	20.00	5.00	0.05	0.74
12	30.00	9.00	0.05	0.86
13	40.00	5.00	0.05	0.35
14	40.00	9.00	0.10	0.38
15	30.00	5.00	0.10	1.12
16	20.00	9.00	0.10	1.22
17	20.00	5.00	0.15	1.14

Analysis of variance showed that the *F* value of the model was 20.66, *p* < 0.001, indicating that the model reached a very significant level. There was no significant difference in the Lack of fit of the model (*p* > 0.05). It means that the non-experimental factors had little influence on the experimental results. And the experimental error was small, which could accurately explain the influence of experimental factors on the response value. There was a big difference between *R*^2^ = 0.9637 and R_pre_^2^ = 0.7270 in the regression model. It showed that the error range of TUDCA/TCDCA predicted by the model was large. Adeq-Precision was 12.821 greater than 4, which indicated that the model could be used for prediction. The model adjusted R-square is 0.9197, which indicated that the model covers the reason of 91.97% response value change.

### Results of Box–Behnken design for incubation condition optimization

The *p* values of variables A and B were less than 0.001 (Table 2), which means that factors A and B were extremely significant, indicating that both the incubation time and temperature have significant influence on the biotransformation of TUDCA, according to the principle that the smaller the *p* value, the more dominant the corresponding influencing factor. The *p* value of C is 0.002, far less than 0.01, indicating that NADP Na_2_ also plays a highly important role in the conversion. The order of the factors influencing the conversion rate was incubation time > temperature > NADP Na_2_.

**Table 2:** Analysis of variance results of regression simulation

Source	Sum of squares	Degree of freedom	Mean square	F-value	*p*-value
Model	1.83	9	0.20	20.66	0.0003
A-Temperature	0.35	1	0.35	35.46	0.0006
B-Incubation time	0.62	1	0.62	63.22	< 0.0001
C-NADP Na_2_	0.22	1	0.22	22.83	0.0020
AB	0.17	1	0.17	17.10	0.0044
AC	2.250E-004	1	2.250E−004	0.023	0.8840
BC	0.018	1	0.018	1.85	0.2156
A^2^	0.20	1	0.20	20.26	0.0028
B^2^	0.20	1	0.20	20.26	0.0028
C^2^	0.013	1	0.013	1.30	0.2925
Residual	0.069	7	9.832E−003		
Lack of fit	0.028	3	9.475E−003	0.94	0.5009
Pure error	0.040	4	0.010		
Total	1.90	16			

**Fig. 4:**
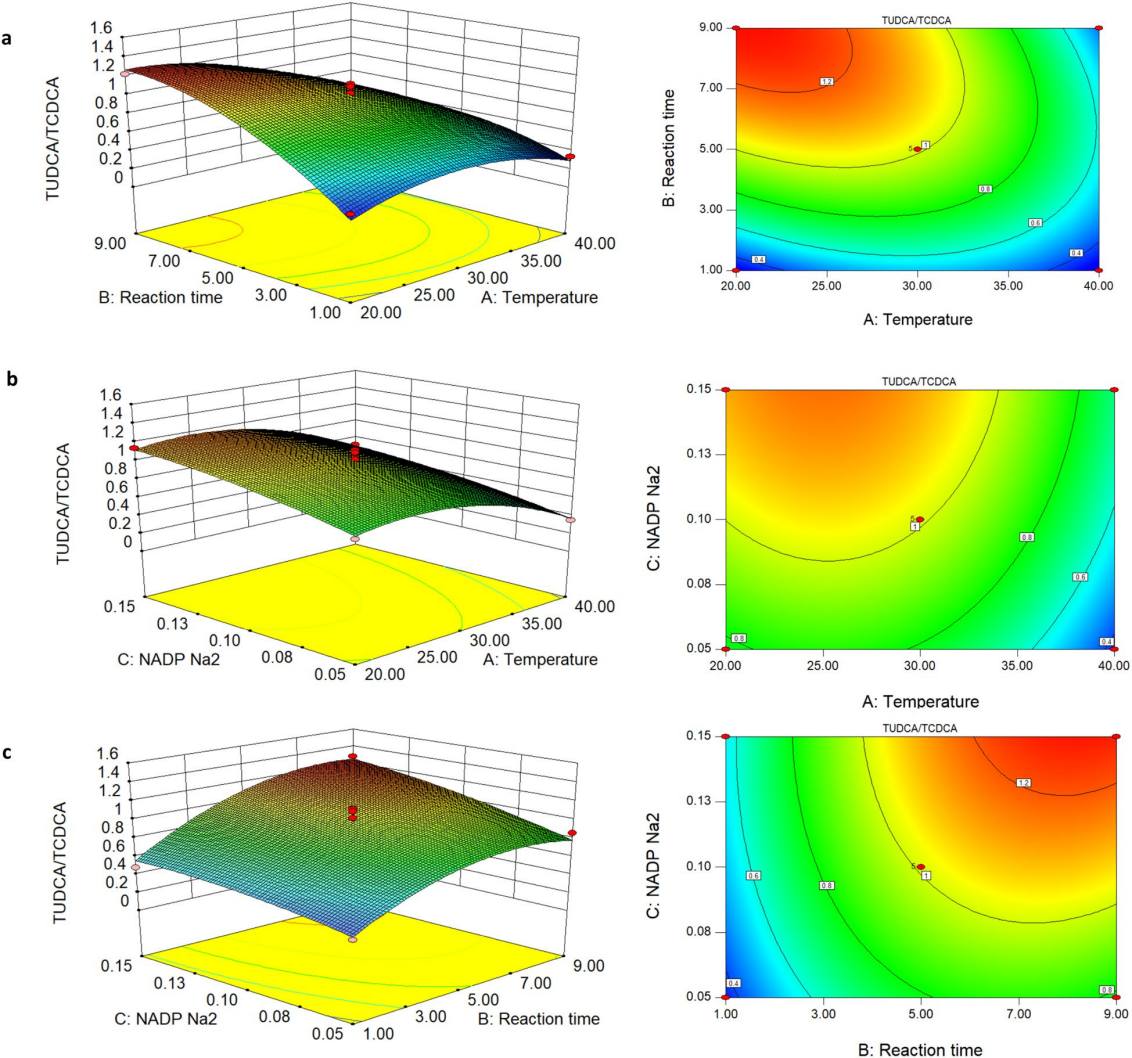
Results of response surface methodology for TUDCA/TCDCA

The significance of the interaction can be reflected by the characteristics of the contour map of the response surface analysis diagram. When the contour map is oval, the interaction is significant; when it is round, it is not significant. Therefore, the importance of the interaction among AB, AC and BC could be intuitively observed. As indicated in Fig. 4, the interaction between temperature and incubation time is the most obvious, which means that the change of temperature significantly affects the incubation time, and vice versa.

According to the results of RSM (Table 3), when three factors (temperature, incubation time, and NADP Na_2_ concentration) were considered together by setting the pH at 7.0 and using 12.00 g/L of substrates, the optimal condition was 25 °C for 5.23 h with 0.08 g/L of NADP Na_2_. The selected conditions were evaluated by performing three repetitions of biotransformation in a 1 mL reaction mixture, and the average value of TUDCA/TCDCA was 1.12, which was close to the predicted value of response surface optimization design. This result confirmed that the optimization model predicted the experimental results well.

**Table 3:** Response surface prediction of top ten reaction conditions

No.	Temperature (°C)	Incubation time (h)	NADP Na_2_ (mg)	TUDCA/TCDCA	Desirability	
1	**25.00**	**5.23**	**0.08**	**1.00**	**0.58**	**Selected**
2	24.92	5.23	0.08	1.00	0.58	
3	25.08	5.21	0.08	1.00	0.58	
4	24.83	5.22	0.08	1.00	0.58	
5	25.18	5.20	0.08	1.00	0.58	
6	25.28	5.21	0.08	1.00	0.58	
7	25.23	5.25	0.08	1.00	0.58	
8	25.20	5.27	0.08	1.00	0.58	
9	25.44	5.21	0.08	1.00	0.58	
10	25.40	5.25	0.08	1.00	0.58	

The top 10 of 32 solutions were listed and the selected one was highlighted in bold

### Preparation and chemical analysis of products

**Fig. 5:**
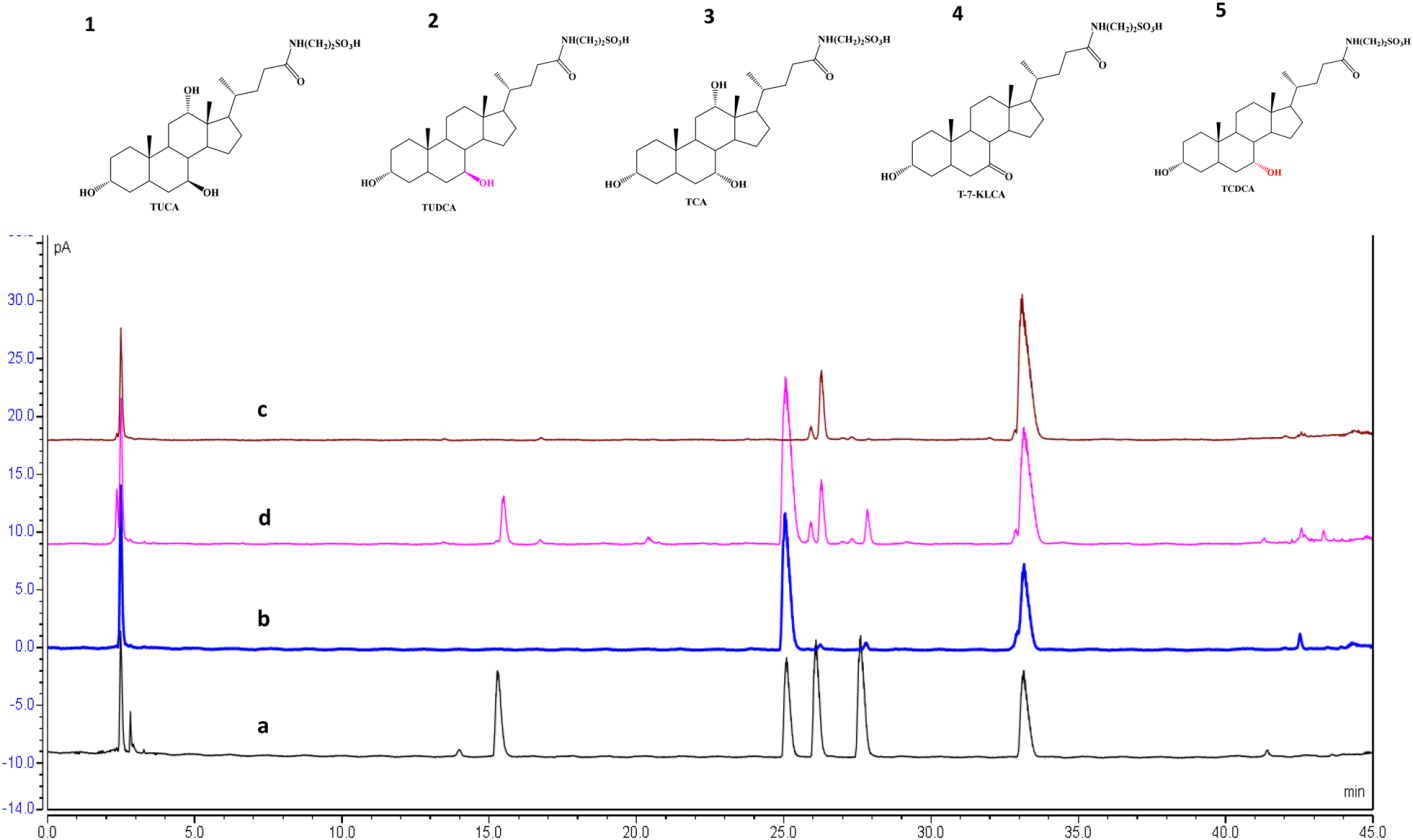
Typical HPLC profiles of bile acids in different samples. **a** Reference standards. **b** Natural bear bile powder. **c** Chicken bile powder (CBP). **d** Biotransformation products from CBP. Compound **1**: tauroursocholic acid (TUCA). Compound **2**: tauroursodeoxycholic acid (TUDCA). Compound **3**: tauro-7-keto lithocholic acid (T-7-KLCA). Compound **4**: taurocholic acid (TCA). Compound **5**: taurochenodeoxycholic acid (TCDCA)

Using the selected optimal condition selected from RSM, 10.99 ± 0.16 g/L of powder product was obtained when incubation of the crude enzymes from CEN-α2β2 with 12.00 g/L of chicken bile powder in 1 L of reaction mixture by addition of 0.08 g/L of NADP Na_2_ at 25 °C for 5.23 h. This powder product contains 36.73 ± 6.68% of TUDCA, and 28.22 ± 6.05% (Table 4). The ratio of TUDCA to TCDCA was 1.30:1.00. The typical HPLC profiles of different samples are exhibited in Fig. 5. As shown, the profile of TUDCA and TCDCA in the biotransformation products was very close to that in the natural bear bile. Yet, the products also contained 2.76 ± 1.24% of tauro-7-keto lithocholic acid (T-7-KLCA), 3.15 ± 0.36% of taurocholic acid (TCA), and 6.34 ± 2.18% of tauroursocholic acid (TUCA) (Table 4; Fig. 5).

**Table 4:** Bile acids in the products

Substrates/products (%)	TUDCA	TCDCA	T-7-KLCA	TCA	TUCA
Chicken bile powder(CBP)	0.00	65.52	0.00	10.21	0.00
Products	36.73 ± 6.68	28.22 ± 6.05	2.76 ± 1.24	3.15 ± 0.36	6.34 ± 2.18

## Discussion

Previously, we had engineered an *E. coli* strain with 7α-HSDH and 7β-HSDH genes that could directional convert TCDCA to a certain ratio of TUDCA. Our study using *E. coli* as host cell demonstrated that all the four combinations of 7α-HSDH and 7β-HSDH, α1β1, α1β2, α2β1, and α2β2, have the capability to convert TCDCA to TUDCA, and fermentation condition has important effect on the conversion efficiency (Shi et al. 2017; Xu et al. 2019).

Different from *E. coli*, *S. cerevisiae* does not produce endotoxin and is regarded as one of the most ideal and safe microorganisms for the production of food and medicinal products. Another advantage of this study is the use of crude enzymes and in vitro biotransformation. It separates the bioconversion process with the yeast cell growth, which facilitates the optimization of the active protein expression and transformation processes, separately, thereby increasing the productivity for industrial application. Besides, the batch preparation of crude enzymes saves the enzyme purification process, so it is more environmentally friendly and green. The disadvantage is that NADP Na_2_ co-factor should be supplemented in vitro reaction mixture.

In this study, CEN-α2β2 is the best combination for TUDCA biotransformation under the given conditions (Fig. 2), which indicates that *S. cerevisiae* CEN.PK2-1C is better than w303-1a as host to express active 7α-HSDH and 7β-HSDH enzymes. Both CEN.PK2 and W303 are commonly used haploid strains for bioengineering with good sporulation efficiency, and CEN.PK2 has a faster growth rate with doubling times of about 80 min for haploid strains (Rogowska-Wrzesinska et al. 2001; Bruder et al. 2016). This fast growth feature is possibly one of the reasons why α2 and β2 expressed in CEN.PK2-1C is more suitable for TUDCA formation.

Both 7α-HSDH and 7β-HSDH have oxidative and reductive properties, and their oxidation or reduction activity is affected by environmental conditions such as temperature and the pH value of the reaction mixture (Yoshimoto et al. 1991; Ferrandi et al. 2012; Lee et al. 2013). Balanced reaction conditions are required to achieve directional biotransformation with a specific ratio of TUDCA to TCDCA. Our results suggested that substrate concentration, reaction time, temperature, the pH value and the NADP Na_2_ concentration have integrated impact on the biotransformation. For example, when 16.00 g/L of substrates and 0.100 g/L of NADP Na_2_ was used, incubation of the reaction mixture at 30 °C for 9 h was good (Fig. 3b). When 12.00 g/L of substrates was used, incubation at 25 °C for 5.23 h with 0.08 g/L of NADP Na_2_ led to the best result (Table 3).

It is worth mentioning that there are two types of 7α-HSDH in nature: one is dependent on NAD^+^, the other is dependent on NADP^+^ (Huang et al. 2019). According to the literatures (Yoshimoto et al. 1991; Ferrandi et al. 2012; Lee et al. 2013), 7α-HSDH from *E. coli* (α2) and *C. sardiniense* (α1), respectively, used NAD^+^ and NADP^+^ as co-factor for enzymatic activity assay. We had separately supplemented β-NAD^+^ and NADP Na_2_ for in vitro biotransformation containing both 7α-HSDH and 7β-HSDH enzymes, but only NADP Na_2_ worked well. So, NADP Na_2_ instead of β-NAD^+^ was used in this study. It is possible that Ec7α-HSDH could also use NADP Na_2_ as co-factor in vitro.

In addition, we found *S. cerevisiae* cell does not allow the main bile acids of chicken bile to pass through its membrane system freely, although it was reported that lithocholic bile acid (LCA) could enter *S. cerevisiae* and accumulated in mitochondria (Beach et al. 2015). Glucanase, NaCl, and Tween 80 had been supplemented into the media to improve the permeability of cell membrane, but only Tween 80 had a little effect, resulting in the production of small amount of TUDCA. This rendered the currently engineered yeast cell unable to directly acts as a whole-cell factory for TUDCA biotransformation. Further modification of yeast cell membrane system will be a potential strategy if whole-cell factories are employed in the future, or using other generally recognized as safe (GRAS) strains such as *Corynebacterium glutamicum* (Fang et al. 2014) as host cells, from the perspective of food and medicinal product safety.

Another issue should be mentioned is that T-7-KLCA, TCA, and TUCA were present in the biotransformation products (Table 4; Fig. 5d). TCA was originated from chicken bile (Fig. 5c) and TUCA was the product of TCA catalyzed by 7α-HSDH and 7β-HSDH. Removal of the OH at C12 of TCA would yield TCDCA, a process possibly catalyzed by 12α-hydroxysteroid dehydrogenase (Tonin and Arends 2018). T-7-KLCA is the intermediate in the biosynthesis of TUDCA from TCDCA (Fig. 1). Directed evolution of 7α-HSDH and 7β-HSDH to enhance the substrate activity and product tolerance may reduce the T-7-KLCA intermediate, as reported for 7α-HSDH (Huang et al. 2019).

In conclusion, through optimizing the *S. cerevisiae* host and gene combinations, and in vitro biotransformation conditions, we have created a green way to make use of cheap and easily available chicken bile powder to produce potential substitute resource for bear bile powder. In order to obtain artificial bear bile that closely matches the chemical composition of natural bear bile, other engineering such as enzyme directed evolution will be helpful.

## Data Availability

The data generated and/or analyzed during this study are available from the corresponding author on reasonable request.

## Abbreviations

7α-HSDH: 7α-Hydroxysteroid dehydrogenase
7β-HSDH: 7β-Hydroxysteroid dehydrogenase
BAs: Bile acids
BBD: Box–Behnken design
CAD: Corona Ultra Detector
GRAS: Generally recognized as safe
LCA: Lithocholic bile acid
PBS: Phosphate buffer saline
RSM: Response surface methodology
T-7-KLCA: Tauro-7-keto lithocholic acid
TCA: Taurocholic acid
TCDCA: Taurochenodeoxycholic acid
TUCA: Tauroursocholic acid
TUDCA: Tauroursodeoxycholic acid
